# Technologies for the Selection, Culture and Metabolic Profiling of Unique Rhizosphere Microorganisms for Natural Product Discovery

**DOI:** 10.3390/molecules24101955

**Published:** 2019-05-21

**Authors:** Saliya Gurusinghe, Tabin L. Brooks, Russell A. Barrow, Xiaocheng Zhu, Agasthya Thotagamuwa, Paul G. Dennis, Vadakattu V. S. R. Gupta, Thiru Vanniasinkam, Leslie A. Weston

**Affiliations:** 1 Graham Centre for Agricultural Innovation, Charles Sturt University, Wagga Wagga, NSW 2650, Australia; sgurusinghe@csu.edu.au (S.G.); tabllewellyn@gmail.com (T.L.B.); rubarrow@csu.edu.au (R.A.B.); xzhu@csu.edu.au (X.Z.); athotagamuwa@csu.edu.au (A.T.); tvanniasinkam@csu.edu.au (T.V.); 2 School of Biomedical Sciences, Charles Sturt University, Wagga Wagga, NSW 2650, Australia; 3 Plus 3 Australia Pty Ltd, P.O. Box 4345, Hawker, ACT 2614, Australia; 4 School of Earth and Environmental Sciences, The University of Queensland, Brisbane, QLD 4072, Australia; p.dennis@uq.edu.au; 5 CSIRO Agriculture and Food, Locked Bag No. 2, Glen Osmond, SA 5064, Australia; gupta.vadakattu@csiro.au

**Keywords:** rhizosphere, soil microbiota, *Brassica napus*, rhizochip, natural products, LC-DAD-QToF-MS, chemical diversity, allelochemicals, in situ isolation, next generation sequencing

## Abstract

Small molecule discovery has benefitted from the development of technologies that have aided in the culture and identification of soil microorganisms and the subsequent analysis of their respective metabolomes. We report herein on the use of both culture dependent and independent approaches for evaluation of soil microbial diversity in the rhizosphere of canola, a crop known to support a diverse microbiome, including plant growth promoting rhizobacteria. Initial screening of rhizosphere soils showed that microbial diversity, particularly bacterial, was greatest at crop maturity; therefore organismal recovery was attempted with soil collected at canola harvest. Two standard media (Mueller Hinton and gellan gum) were evaluated following inoculation with soil aqueous suspensions and compared with a novel “rhizochip” prototype buried in a living canola crop rhizosphere for microbial culture in situ. Following successful recovery and identification of 375 rhizosphere microbiota of interest from all culture methods, isolates were identified by Sanger sequencing and/or characterization using morphological and biochemical traits. Three bacterial isolates of interest were randomly selected as case studies for intensive metabolic profiling. After successful culture in liquid media and solvent extraction, individual extracts were subjected to evaluation by UHPLC-DAD-QToF-MS, resulting in the rapid characterization of metabolites of interest from cultures of two isolates. After evaluation of key molecular features, unique or unusual bacterial metabolites were annotated and are reported herein.

## 1. Introduction

The release of carbon containing root exudates and/or rhizodeposits by terrestrial plants influences the soil environment, attracting and sustaining microbial populations that are more abundant than those from root-free soils [1]. Rhizosphere associated microorganisms typically form complex interactions that impact on plant growth and nutrition [2,3]. The relative influence of these communities on plant health is great, with the microbiome of the rhizosphere and endosphere collectively referred to as the plant’s second genome. This genome is significantly larger than that of the plant, and the associated metabolic activity of its microbial members is influenced and coordinated by the plant to assist in its development and defence. Metabolic activities in the rhizosphere include chemical signals secreted by roots (e.g., allelochemicals), production of plant growth hormones and metabolites associated with plant stress as well as defence against pathogens, weeds, insects and other pests [4]. Selection pressures associated with below-ground interactions frequently drive the evolution of complex defence systems in soil microorganisms, influencing their survival and manifesting in the production of antibiotics or toxins [5,6], metabolites which can be repurposed for biomedical or agricultural uses [7], or facilitating adaptations which allow the expression of diverse biosynthetic pathways [8,9]. However, serious limitations in the availability of high-throughput technologies for microbial isolation, characterization of isolates and structural elucidation of novel bioactive secondary metabolites have limited the number of natural products discovered over the last few decades [10].

In recent years culture independent profiling of the soil rhizosphere microbiome using metagenomics approaches has revealed that the abundance and diversity of the total microbial community is far greater than what is represented in the cultivable component [11,12,13,14]. It has been suggested that up to 1% of soil bacteria are readily cultivable; however, in the rhizosphere where nutrient levels are higher, the rate of recovery may increase to 10% [15,16]. Agar-based culture media typically generate biased subsets of microflora, or “microbial weeds” and often do not recover slow-growing taxa [17]. However, the rate of recovery can be increased through optimisation of culture conditions by reduction of nutrient concentration, modulation of temperature and increased incubation periods [18,19,20]. Further refinements by altering the culture substrate from agar to gellan gum and targeting of small slow-growing colonies have resulted in the recovery of both novel and dominant soil taxa not previously cultured [18,21].

Recently, the recovery of cultivable soil bacterial populations was improved by up to 50% of the total microbial taxa with the use of an in situ culture apparatus, the “iChip” [22]. Possessing micro growth chambers permitting the diffusion of soluble nutrients present in the rhizosphere to stimulate bacterial growth in situ, the iChip typically promotes colonization of fastidious microorganisms, which can be further subcultured in vitro. In situ culture is also thought to alleviate habitat constraints posed by in vitro culture by facilitating consistent exposure to naturally occurring small molecules or nutrients present in the soil environment to initiate enhanced cell division or sporulation [23]. Improved recovery rates using the iChip apparatus have resulted in the identification of novel species, thereby expanding the limits of reported microbial diversity, and identification of associated genes encoding for chemical diversity, as evidenced by the discovery of the novel antibiotic teixobactin from the bacterium *Eleftheria terrae* [15,22].

The use of microbial phylogenetic marker gene analyses [24], advanced proteomic and metabolomic approaches [25], and co-culture experimentation with diverse species [26] have further revealed complex plant-microbiome interactions. Since many microorganisms devote up to 20% of their genome to the production of secondary metabolites [27] application of such technologies offers great potential for expanding chemical diversity. Recent advances in mass spectrometry (MS) have also dramatically contributed to the capacity to screen microbial metabolites by coupling MS to gas or liquid chromatography enabling rapid identification of chemically diverse metabolites. The use of nanospray desorption electrospray ionization mass spectrometry (nanoDESI-MS) technology has allowed researchers to detect organic molecules including quorum sensing agents, antibiotics, glycopeptides and oligosaccharides secreted directly by living bacterial colonies [19,28,29]. Both targeted and non-targeted metabolomic analysis of organismal extracts supported by mass spectrometry has contributed greatly to our understanding of the complex relationships existing between plants and rhizosphere microorganisms and assisted identification of both microbial and plant secondary products associated with their interactions [25,30,31,32]. Such approaches have contributed significantly to the discovery of new natural products and pathways [33,34].

**Figure 1:**
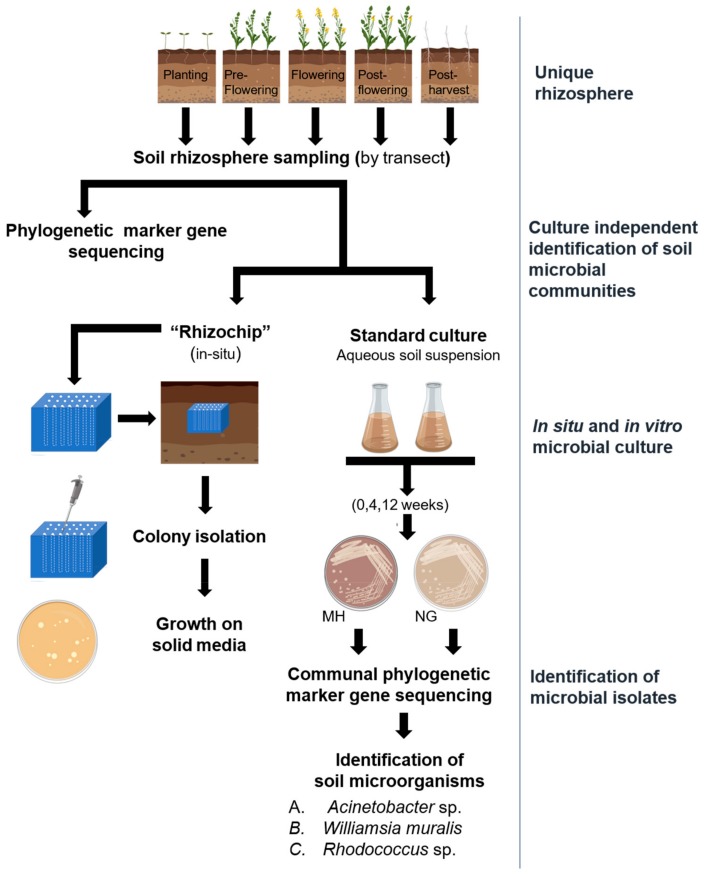
Schematic representation of the workflow associated with the isolation and culture of slow-growing microbial populations from the canola rhizosphere. NG: Nutrient broth gellan, MH: Mueller Hinton.

In the present study, we tested the hypothesis that utilisation of non-standard soil microbial culture methods enables successful isolation of rare or slow-growing microbial isolates producing bioactive secondary metabolites. To this end, we used phylogenetic marker gene sequencing to characterise the dominant microbial taxa present in a living canola (*Brassica napus*) rhizosphere, as canola is known to support a diverse microbiome [9]. The recovery of microbial taxa between the ‘rhizochip’ in situ culture method and standard in vitro culture techniques was compared (Figure 1). We report on the results of several case studies to support the assertion that the rhizosphere represents an important and emerging resource for the discovery of unique natural products for both agrichemical and pharmaceutical needs.

## 2. Results

### 2.1. Phylogenetic Marker Gene Sequencing for Identification of Bacterial and Fungal Taxa in the Canola Rhizosphere

A preliminary study to evaluate the presence of bacterial and fungal taxa within the rhizosphere at various growth stages of the canola crop was conducted using phylogenetic marker gene sequencing at five time points ranging from planting to harvest of the canola crop. In all samples collected over the growth season, bacterial operational taxonomic units (OTUs) were dominated by Actinobacteria (38.7 ± 7.5%) followed by Proteobacteria (29.1 ± 7.0%) at the phylum level while fungal OTUs were dominated by Ascomycota (65.6 ± 15.8 %) and Basidiomycota (14.19 ± 8.1%), a finding similar to that reported by Bisset et al. in a broad-ranging study of Australian soils [35] (Supplementary Materials Figure S1A,B).

### 2.2. Identification of Selected Bacterial Colonies by Sanger Sequencing of 16S rRNA

With the greatest diversity in rhizosphere microbial communities for both bacterial and fungal OTUs typically observed at crop maturity, our efforts for the isolation of novel and slow growing microorganisms were concentrated on rhizosphere soil collected at harvest, with corresponding rhizochip apparatus retrieval from the field site also at harvest. The isolation and culture of bacterial and fungal cultures were performed with two general non-selective, and non-differential culture media. Nutrient broth solidified with gellan gum (NG) and Mueller Hinton (MH) solidified with agar were selected as solid media to maximise the isolation of microorganisms, with the formulation of the latter containing nutrients preferable for the isolation of sporulating bacteria [36]. The total number of colony forming units (CFUs) peaked at 4 weeks (as opposed to 12 weeks) of incubation of the aqueous soil suspension to likely enhance sporulation of organisms after inoculation on either NG or MH solid media and CFUs did not significantly different between media (Supplementary Materials Figure S2). A total of 375 isolates was generated following inoculation on solid media, with 32 originating from the rhizochip-based isolation strategy and the remaining 343 isolated from the aqueous suspension based isolation of soil microorganisms. As both media chosen for culture were not selective for fungal isolation, it was not surprising that bacterial isolates predominated.

### 2.3. Genomic Identification of Isolated Microbial Cohorts Through Standard- and Rhizochip-Based Culture Methods

**Figure 2:**
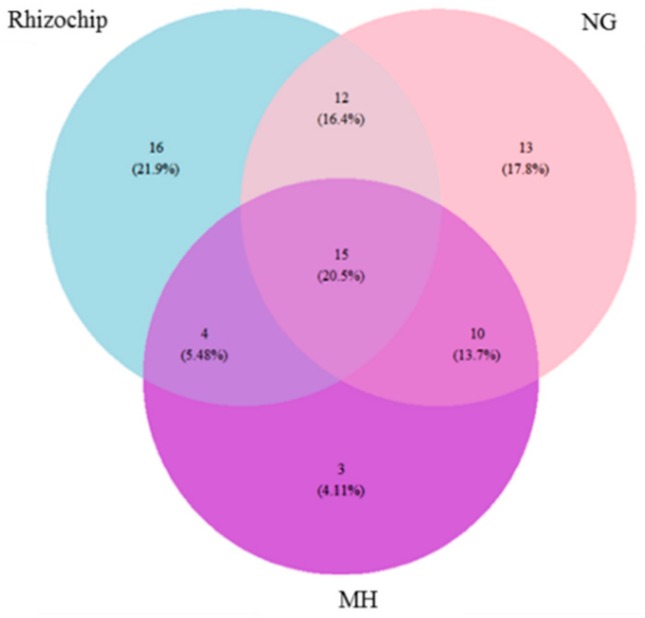
Venn diagram demonstrating unique and shared operational taxonomic units (OTUs) between three microbial isolation methods at the order level featuring the rhizochip and solid media NG (gellan gum) and MH (Mueller Hinton).

Phylogenetic marker gene sequencing was employed for rapid identification of isolates and enabled clear differentiation between the isolation strategies attempted (Supplementary Materials Figure S3). The composition of bacterial OTUs at the phylum level obtained through in situ rhizochip and in vitro culture was reasonably similar to those identified through phylogenetic marker gene sequencing of the rhizosphere soil, with Firmicutes and Proteobacteria being dominant phyla. However, Actinobacteria were not represented in the cultured cohort when ranked over >1% absolute abundance, suggesting that they were not easily cultivable using the culture systems employed. The rhizochip was however successful in isolating 16 unique OTUs while NG (gellan gum) and MH methods isolated 13 and 3 unique OTUs respectively, at the order level (Figure 2). A total of 15 OTUs were shared between the three isolation methods. Rhizochip and NG shared the highest number of OTUs at 28. Bray Curtis dissimilarity index revealed that the order Bacillales contributed most significantly to the dissimilarity between the rhizochip and NG (11.3%) and NG vs. MH solid agar (14.4%) groups (Supplementary Materials Table S1). In contrast, the order Actinomycetales contributed most to the dissimilarity between the rhizochip and MH groups. The complete list of OTUs identified using three culture methods is presented in (Supplementary Materials Table S2).

### 2.4. Identification of Selected Bacterial Colonies by Sanger Sequencing of 16S rRNA

The identity of selected microbial isolates of interest was confirmed through Sanger sequencing targeting the 16S rRNA region. The 15 entries submitted included five isolates obtained through use of the rhizochip, while a further five were obtained through MH culture and the remainder through isolation on NG. The identification of the isolates to strain level and their agroecological relevance in corresponding literature citations are presented in Table 1.

### 2.5. Metabolic Profiling of Selected Organisms

Of the 375 microorganisms identified in this study, three were selected as case studies for further metabolic profiling based on such factors as ability to be cultured on solid and liquid culture media, novelty of the genera, morphology and/or pigmentation. The isolates selected were assigned the colony identification numbers 240, 241 and 321.

**Figure 3:**
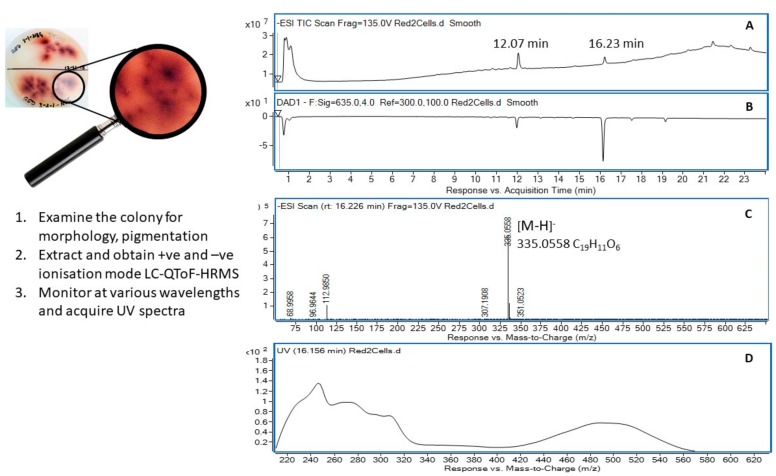
(**A**) *Acinetobacter* sp. #321, negative ion (−ve) TIC chromatogram from C18 reverse phase chromatography; (**B)** Corresponding absorbance chromatogram at 635 nm; (**C)** Mass spectrum (−ve ion) of the compound eluting at 16.2 min; (**D**) UV-vis absorbance spectrum (200–635 nm) of the metabolite eluting at 16.2 min.

**Figure 4:**
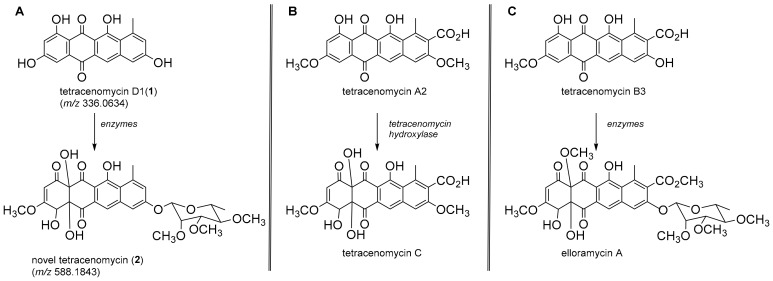
(**A**) Proposed metabolites (**1**) and (**2**) produced by *Acinetobacter* sp.; (**B**) transformation involving the conversion of tetracenomycin A2 to tetracenomycin C [48]; (**C**) transformation involving the conversion of tetracenomycin B3 to elloramycin A [47].

Colony ID 321 was identified as a Gram negative *Acinetobacter* sp. (98.8% sequence similarity). This bacterium demonstrated the capacity to produce a red pigment that leached into the media when grown on NG and readily grew in a nutrient broth culture. Centrifugation of the liquid culture and immediate extraction of the cellular material with 95% ethanol produced a bright red solution in which the cells were suspended. Filtration followed by removal of the solvent under a stream of nitrogen at 35 °C produced a dark red gum that was dissolved in acetonitrile and subjected to UHPLC-DAD-QToF-MS analysis (Figure 3). High resolution mass assessment of the compound eluting at 16.2 min (Figure 3A,B) revealed it possessed a molecular formula corresponding to C_19_H_12_O_6_ (Figure 3C) and displayed a strong chromophore that was considered responsible for the pigmentation observed in the *Acinetobacter* sp. The UV-visible absorbance spectrum (Figure 3D) was remarkably similar to those belonging to members of the tetracenomycin family and after a literature search, the molecule eluting at 16.2 min was annotated as tetracenomycin D1 (**1**) (Figure 4A) [47].

A second chromophoric compound was recognised in the absorbance chromatogram (Figure 3B) eluting at 12.1 min and it displayed an ion in the negative ionisation mode at 587.1776 ([M − H]^−^) supporting a molecular formula of C_29_H_32_O_13_. This molecular formula was further supported by the appearance of [M + Na]^+^ and [2M + Na]^+^ adduct ions at 611.1720 and 1199.3593, respectively, in the positive ionisation mode QToF-MS experiment. The corresponding UV-visible absorbance spectrum revealed a hypsochromic shift of an absorption at λ_max_ 500 nm observed in tetracenomycin D1 (**1**) to λ_max_ 445 nm consistent with a deconjugation of a tetracyclic system and consistent with the hypsochromic shift observed for the transformation in similar systems [47]. We suggest that the molecule eluting at 12.1 min (**2**) is likely a novel structure representing a rhamnoside of the hydroxylated version of tetracenomycin D1. Further work is underway to confirm this tentative assignment; however, the oxidation has precedence, amongst others, in the conversion of tetracenomycin A2 to tetracenomycin C [48] (Figure 4B). The rhamnose glycoside is also common among related metabolites, for example in the conversion of tetracenomycin B3 to elloramycin A (Figure 4C) [47].

**Figure 5:**
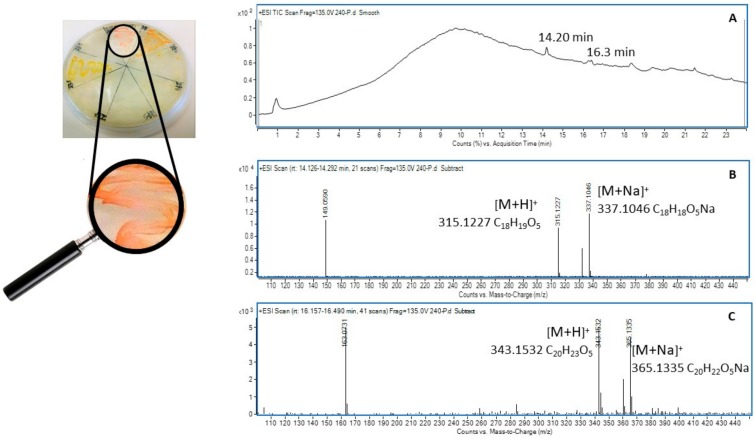
(**A**) *Williamsia muralis* str. 9571414, positive ion (+ve) TIC chromatogram from C18 reverse phase chromatography; (**B**) Mass spectrum (+ve ion) of the compound eluting at 14.2 min; (**C**) Mass spectrum (+ve ion) of the compound eluting at 16.3 min.

Colony ID 240 was identified as the Gram positive Actinobacterium *Williamsia muralis* str. 9571414 (99.9%) based on 16S rRNA analysis. *Williamsia muralis* isolated from the rhizosphere soil was characterised by smooth pink pigmented colonies (Figure 5) that grew well on 2% gellan gum containing nutrient broth media and in nutrient broth liquid culture. Centrifugation of the liquid culture provided a pellet of pink cells that were immediately extracted in 95% ethanol to return a light yellow solution with the pink pigment remaining unextracted in the cellular suspension. Filtration followed by removal of the solvent under a stream of nitrogen at 35 °C produced a yellow gum that was taken up in acetonitrile (2 mL) and subjected to UHPLC-DAD-QToF-MS analysis.

**Figure 6:**
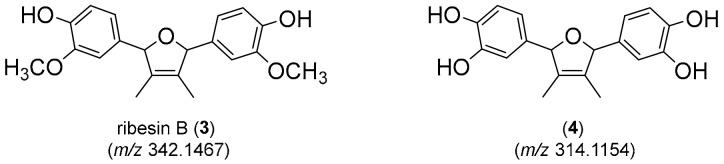
Structures of ribesin B (**3**) and its desmethyl analogue (**4**).

Consideration of the molecular formulae for the two metabolites identified in Figure 5B/C suggested they were analogs, differing by two methylations on the higher mass molecule (*m*/*z* 342) compared to the lower mass molecule (*m*/*z* 314). This assertion was supported by the major fragment ion observed in each occurring at *m*/*z* 163 and *m*/*z* 149 respectively, supporting a homodimeric structure. An extensive review of the chemical literature cross referencing these data identified the antioxidant ribesin B (**3**) [49], a 7,7′-epoxylignan from the plant *Ribes nigrum* (black currant) as a plausible structure for the molecule eluting at 16.3 min with *m*/*z* 342. This necessitated the molecule eluting at 14.2 min with *m*/*z* 314 to be the desmethyl analog **4** (Figure 6).

The third and final colony selected for metabolomic analysis was identified as another gram positive Actinobacterium, *Rhodococcus* sp. str. 5/14 (99.85% sequence similarity) and was selected based on the yellow-orange pigmentation of the colony and the known capacity of members of the genus to catabolize herbicides [50]. Despite a highly pigmented pellet being obtained after centrifugation of the culture broth, the ethanolic extract was only slightly coloured and upon subsequent analysis by UPLC-DAD-QToF-MS did not reveal any molecular features of interest and further analysis was abandoned.

## 3. Discussion

Cultivable environmental microbes have been a major source of lead molecules for the development of therapeutic drugs and to lesser extent agricultural products or amendments following the discovery of Penicillin from *Penicillium rubens* in 1929 [51,52]. However, it is now becoming evident that high rediscovery rates of similar bioactive molecules from readily cultivable microbes is impacting the development of lead molecules with novel modes of action, highlighting the need to explore previously unexplored molecules produced by highly competitive [53] and rare microbes surviving under harsh environmental conditions, frequently synthesizing unique chemical defence compounds [54]. Australian soils, specifically those generally considered to be degraded, have been previously studied across various environmental gradients to assess the impact of soil types, moisture and land-use on the microbial diversity to better understand soil functions and ecosystem services provided by microbial communities often surviving under harsh environmental conditions [35]. Results suggest that the microbiome is clearly impacted by environmental and land use patterns. Crop phenology has also been shown to impact the composition of soil microbial communities that are also influenced by the microenvironment provided by crop roots and their exudates [1,55]. Interestingly, our findings showed that dominant phyla of both bacteria and fungi commonly noted in Australian soils were also represented proportionally in canola rhizosphere soils, as assessed by phylogenetic marker gene sequencing. Proteobacteria and Actinobacteria generally dominated bacterial phyla, while Ascomycota and Basidiomycota dominated fungal phyla, with a notable exception at the time of flowering associated with an increased abundance of Firmicutes, an observation also made previously in a similar canola rhizosphere [56].

Sampling of soil and the subsequent use of an array of standard culture techniques has traditionally been used to isolate soil microbes, some of which have proven to be functionally useful. However, an enhanced understanding of the interactions that exist between plants and their microbial partners will allow researchers to select more appropriate sampling criteria for these partners. The rhizosphere, the region of soil immediately surrounding the roots, is considered the next frontier in chemical ecology [57] and it has been recognised that knowledge about microbiome interactions (signalling/inhibition/syntrophy) is critical to improve the understanding of rhizosphere functions, responses and properties [58]. These remain largely unexplored because of the magnitude of microbial diversity, and the difficulty encountered in recovering and culturing soil microbiota [59].

Fluctuation in soil microflora has generally been reported over the crop growing season [12] as the immediate microenvironment is altered due to root exudation and turnover, and the availability of soil nutrients, particularly following the incorporation of plant residues [26], or due to direct contact with living roots [5]. Rate of root exudation and turnover can also change dramatically at different phases of the plant’s lifecycle or under different growth conditions [32]. Canola is a Brassicaceous plant that contains plant-produced secondary products, glucosinolates and isothiocyanates, and is also known for its unique rhizosphere environment which harbors a diverse microflora [54,60]. The production and persistence of such metabolites varies with plant growth stage and hence rhizosphere and root associated microbial diversity also varies with plant developmental age [9]. This study was specifically designed to (1) screen temporal changes in microflora in the canola rhizosphere over time (2) demonstrate the potential to isolate and culture rare or unique soil microbiota, and (3) further explore chemical diversity produced by associated microflora in the canola rhizosphere.

Seasonal variation in rhizosphere microbial community profiles has been previously reported in canola, with respect to plant phenology, through evaluation of ribosomal DNA restriction analysis profiles [9]. However, while differences in community profiles were observed over time in previous work, the total microbiome was not investigated. Our findings suggest that fungal and bacterial diversity was higher at the time of canola harvest and are in agreement with previous reports on the temporal variation of microbial communities with respect to crop phenology [61,62]. Temporal variation is frequently associated with differential root exudation [8,62,63], edaphic factors [64,65] or use of agricultural practices including tillage [63,66].

The recruitment of specific microorganisms is postulated to occur early on in plant growth and development, following the initiation of root exudation [67]. Reduction in soil microbial diversity noted directly after planting may be associated with soil disturbance within the various horizons, impacting microbial compositions [68], with other immediate changes associated with the establishing crop, variation in soil temperature and/or moisture availability. However, following crop establishment, plant secondary metabolites, including the flavonoids and canola-produced glucosinolates, are known to function as root signalling molecules and can facilitate microbial attraction or deterrence and consequent interactions [30]. Over time, the production of root exudates by the establishing crop may allow for enhanced microbial numbers and potentially microbial diversity.

The rhizochip, a novel in situ microbial culture apparatus, was developed by our research team for the isolation of unusual or slow-growing microbiota from plant rhizospheres, with some similarities to the patented iChip [22]. In the case of the rhizochip, each of the 60 individual micro-chambers was potentially exposed to numerous organisms as the soil solution diffused randomly throughout the apparatus, in contrast to the iChip which was assembled with several hundred miniature diffusion chambers, each inoculated with a single environmental cell. We compared the rhizochip in situ cultivation method with standard soil rhizosphere microbial isolation methods based on inoculation of soil suspensions in two non-differential solid culture media (NG and MH), through subsequent examination of microbial isolates identified using phylogenetic sequence analysis. Interestingly, sequencing analysis revealed that the greatest number of unique OTUs (21.9%) was isolated using the rhizochip in situ culture method (Figure 2). Our findings further confirm that provision of microsites through individual micro-chambers promotes the isolation of otherwise inaccessible microorganisms [22]. Given that Australian agricultural soils are generally low in organic matter and fertility, associated microflora may be more reliant on naturally occurring growth promoters rendering them less responsive on nutrient rich media [69,70]. Therefore, such in situ technologies offer enhanced possibilities for the isolation of rare and slow-growing soil organisms in degraded Australian soils.

Specifically, the rhizochip prototype we developed was created to allow enhanced isolation of fastidious, or slow growing soil microorganisms. A similar strategy was first described by Nichols et al. [22] where the iChip, constructed with 384 channels, was inundated with a microbial suspension which was subsequently capped with agar and cultured in situ in a field location. In contrast, the rhizochip prototype culture apparatus (60 channels) has reduced screening capacity and utilizes pre-infiltrated agar channels which are inoculated with a microbial suspension prepared from rhizosphere soil collected. While the iChip was designed to recover single colonies or isolates per compartment, our methodology does not limit colonization by single species. The successful expansion of colonies using this method yielded numerous bacteria that are recognised for their importance in crop protection and agroecology (Table 1). For example, *Pseudomonas costantinii* in the class Gammaproteobacteria is a known fungal pathogen affecting mushroom production [37], *Paenibacillus polymyxa* is a bacterial species within the class Bacilli with insecticidal and herbicidal properties [38] and *Variovorax paradoxus* exhibits potent plant growth promoting activity [42]. Additionally, the *Acinetobacter sp.* recovered is known for its use as a model for environmental and biotechnological applications in reclamation [40] and *Arthrobacter nicotinovorans* is recognised for its abilities to degrade herbicide residues in soil [46].

Standard techniques for the culture of soil microbiota were also evaluated in this study; such techniques are often geared towards recovery of a small subset of the total soil community. Fast growing organisms often predominate when soil aqueous suspensions are inoculated on standard growth media. To minimize their predominance, aqueous soil suspensions were incubated for an extended period (4 or 12 weeks), to potentially increase the rate of sporulation of slow growing or rare microorganisms [36,71]. Interestingly, following extended incubation, the greatest number of CFUs was obtained from soil extracts subjected to 4 weeks of incubation in contrast to immediate inoculation on solid media or incubation for 12 weeks. This finding is in agreement with previous reports showing extended incubation times under oligotrophic conditions enhanced the cultivable component of the microbial assemblage, but extended periods of incubation (i.e., 12 weeks) result in reduced spore viability [72]. While employing novel isolation methods may not entirely address the challenges surrounding isolation of bacteria from diverse soil types exhibiting variability in nutrients and growth factors, such methods may increase the likelihood of isolation of slow growing or fastidious microorganisms when used in combination with specialised culture substrates, as our findings have suggested.

As discussed by Hamaki et al. [73] the use of soil-based modified growth media reduces the likelihood of imposed bias on the diversity of microbiota isolated from rhizosphere soil. However, media containing soil extracts are not easily reproducible due to variation in soil composition over time and location. Thus, with the aim of standardising laboratory culture methods, commercially accessible gellan gum with nutrient broth and Mueller Hinton based media were used in this study. We therefore investigated the efficacy of both media formulations to isolate slow-growing or fastidious soil microbiota with the desire to explore their chemical diversity. Mueller Hinton was also included in colony isolation and expansion as it was previously reported to promote the sporulation of *Bacillus* spp. [36]. Of the isolated bacteria characterised, a smaller subset were successfully subcultured. This is possibly due to culturing recalcitrance, a limitation previously described by Kaeberlein et al. [74]. The methods employed in this study also resulted in recovery of a very small number of fungal isolates, specifically two colonies. This may be due to media bias, as the media selected for this study were chosen for their reported capacity to promote bacterial isolation. Further experimentation to improve fungal recovery is likely to necessitate specific media suitable for fungi such as potato dextrose agar [71,73].

Following identification, of a selection of bacterial isolates was subjected to a non-targeted metabolic profiling approach, employing UHPLC-DAD-QToF-MS, to comprehensively evaluate the metabolites present in solvent extracts of three bacterial isolates. The first isolate studied was identified as an *Acinetobacter* species (colony ID 321, a producer of red pigmented metabolites in liquid culture. The genus is a strictly aerobic, soil borne, Gram negative coccobacilli that has been reported to be an emerging nosocomial multi-drug resistant (MDR) pathogen [75]. It is known to tolerate dry conditions and has been identified in Australian soils [35,76]. Currently The MDR properties of *Acinetobacter* have been attributed to their ability to produce biofilms induced by quorum sensing mechanisms [77]. The study of associated metabolites was hypothesised to give further insights into its competitiveness against other microorganisms. Analysis of the pigmented cellular constituents allowed the tentative assignment of associated red metabolites as tetracyclic molecules of the anthracycline class, namely tetracenomycin D1 (**1**) and a novel hydroxylated rhamnoside (**2**) (Figure 4). To our knowledge, this represents the first report of this class of molecule from *Acinetobacter* although the type II polyketide biosynthetic machinery noted for production is known to exist in this genus. [78]. The observation of further unidentified and minor metabolites in the UV chromatogram (Figure 3B) highlights the potential of this bacterium to produce additional metabolites warranting further study.

The second isolate studied was identified as a *Williamsia* sp, potentially *W. muralis*. This genus of gram positive aerobic Actinobacteria was recently created to account for a distinct group of organisms that fit phylogenetically between the genera of *Rhodococcus* and *Gordonia* [79]. The genus was initially characterised in 1999 with *W. muralis* first isolated [80], however over 10 species have now been identified, including *W. marianensis* isolated from deep sea sediments at 11 km depths in the Mariana trench [79]. *W. muralis* is also considered to be rare, and was cited as infrequently encountered in soil [54]; recently, it was reported as an endophyte of grey box eucalyptus (*Eucalyptus microcarpa*), a species commonly encountered in southeastern Australian woodlands and native grasslands [18]. Its presence in the plant phyllospshere is indicative of its capacity to tolerate desiccation and UV radiation [81]. The novelty of this bacterium highlighted it as a candidate for metabolomic analysis and the tentative identification of its associated tetra-substituted 2, 5-dihydrofurans lignans (**3**) and (**4**) were made by correlating high resolution mass data with a natural product database. The production of lignans is well known in plants, as exemplified by the production of ribesin B (**3**) by *Ribes nigrum* [49]. Plant-associated microflora have been recently noted as an alternative source of such lignan and neolignan metabolites [82] and the identification of the known lignan **3** and the novel metabolite **4** support this finding.

The final isolate studied from a metabolomic perspective was identified as another Gram positive Actinobacteria, *Rhodococcus* spp. isolate 241. The polyspecific genus includes numerous species of industrial importance for steroid production [83], biodesulfurization of diesel [84] and biodegradation of nitriles [85]. This genus contains numerous species with active polyketide synthase genes raising the possibility for the production of extensive secondary metabolites through the polyketide pathway [86]. Unfortunately, extracts of the *Rhodococcus* species isolated in our study did not produce molecular features of interest following metabolic profiling.

In summary, cultivable environmental microbes have been a major source of lead molecules for the pharmaceutical and agrichemical industries. However, the discovery of metabolites with novel modes of action can logically be facilitated by further evaluation of highly competitive and rare microbes surviving under harsh environmental conditions, specifically those utilizing unique chemical defences. In Australia, plants and associated rhizosphere microbiota are exposed to particularly harsh environmental conditions. This study has explored the opportunity for recovery and subsequent culture of rare or slow-growing microbiota from a canola rhizosphere using both standard and novel in situ culture techniques, and culture dependent and independent techniques for their identification. The rhizochip has proven to be useful for recovery of unique soil microbiota, along with prolonged incubation of aqueous soil extracts followed by inoculation on standard low nutrient solid media based on gellan gum. Case studies performed with microbial isolates of interest have shown that rapid characterization of microbial metabolites is possible using metabolic profiling by UHPLC-DAD-QToF-MS, facilitating the discovery of both novel and interesting natural products.

## 4. Materials and Methods

### 4.1. Establishment of the Canola Field Trial

A canola field trial was established at the Graham Centre field site, Wagga Wagga, NSW, (35°02′39.8″ S 147°21′54.3″ E) and was planted in 2016 as a randomised complete block design with five replications as part of a long term crop rotation study. The soil type at this site was characterised as red Sodosol at pH 6.2. The crop was produced using standard agricultural practices.

### 4.2. Soil Sampling

Soil samples were collected from each of the five replicated plots of GT-50 hybrid canola using a soil corer (Nutri-Tech Solutions, Yandina, QLD, Australia) with a diameter of 5 cm to a depth of 10 cm from the soil surface. Five rhizosphere soil samples were taken from each of the plots for the purpose of microbial genomic DNA extraction and for generating soil suspensions for later use in both in vitro and in situ culture of soil microorganisms. Soil sampling for the purpose of DNA extraction was performed throughout the growing season at corresponding growth stages of the canola crop (Table 2). One gram aliquots of composite soil samples were stored at −80 °C until subsequent genomic DNA extraction.

### 4.3. In Vitro Culture of Soil Microorganisms Obtained through Soil Suspensions in Various Culture Media and Solidifying Agents

A one gram aliquot of rhizosphere soil previously collected and combined at each growth stage of the canola crop was suspended in 10 mL of sterile distilled water to produce a 10% (*w*/*v*) soil suspension. Petri dishes containing 0.013%, 0.13% and 1.3% (*w*/*v*) nutrient broth solidified with 2% gellan gum (Gelzan™, Sigma Aldrich, Castle Hill, NSW, Australia) or 1× Mueller Hinton (Sigma Aldrich, NSW) solidified with 2% agar were inoculated as lawn cultures either immediately after preparing the suspension or following 4 or 12 week incubation at 25 °C to promote spore formation and reduce the emergence of fast growing microorganisms. Five petri dishes were inoculated at each time point and the resulting cultures were maintained for 4 weeks at 25 °C at which point final colony numbers were recorded, growth characteristics recorded, sporulation status evaluated and colonies exhibiting pigment production enumerated. Single colonies were further isolated and cultured in 0.13% nutrient broth solidified with gellan or in Mueller Hinton solidified with agar.

### 4.4. In Situ Culture with a Prototype Rhizochip Apparatus and Isolation of Microbial Colonies

**Figure 7:**
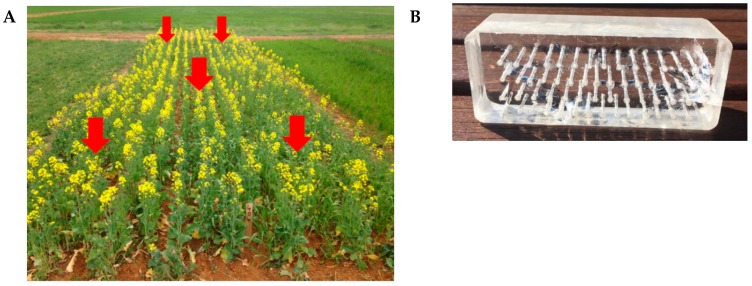
Locations of rhizochips within the canola plots with red arrows pointing to sites of burial (**A**) and representative image of the rhizochip with predrilled micro-chambers (**B**).

A prototype in situ soil microbial culture apparatus, the rhizochip, was constructed to promote establishment and growth of slow growing soil microorganisms. Briefly, 60 micro-chambers (1 mm diameter, 35 mm depth) were created in a clear acrylic block (dimensions: 110 × 40 × 35 mm) to facilitate microbial growth. Following sterilisation with 70% ethanol for 12 h, each of the micro-chambers was impregnated with 0.013% nutrient broth (Oxoid, UK) solidified with 2% gellan gum (Sigma Aldrich, NSW) [59]. A 1% (*w*/*v*) soil suspension was prepared in sterile distilled water (100 mL) and filtered with an autoclaved Whatman No. 1 filter to remove soil particles. The resulting microbial suspension was then used to inoculate the top surface of the micro-chambers in an acrylic block by briefly immersing the block in the suspension. A sterile 0.20 µm nylon membrane (Sigma-Aldrich, NSW) was then placed over the surface of the micro-chambers to facilitate gas and nutrient exchange. The acrylic block was then placed in a sterile plastic container previously sterilized with 70% ethanol for 12 h and the entire assembly was sealed with a lid containing multiple perforations (60 total with approximately 2 mm diameter pore size) to allow for soil moisture diffusion (Figure 7). Five rhizochips were buried approximately 10 to 20 cm below the soil surface in the A horizon of soil profile, between the rows of canola before seeding and were retrieved at various growth stages during canola growth.

Following disassembly of the rhizochip, retrieval of microbial colonies from individual microchambers was performed by individually isolating a small volume of the fresh solid media with a sterile wooden pick to a depth of approximately 10 mm and transferring to a sterile petri dish containing 0.13% nutrient broth (with 2% gellan) (NG). Plates (approximately 20 to 30 per apparatus) were then incubated at 25 °C and 30 °C for a maximum of 8 weeks to facilitate the growth of slow growing bacterial and fungal colonies. The emerging single colonies were then individually isolated on solid plates. Slow growing colonies were defined as those which became visible following a minimum of 14 days of incubation at two incubation temperatures. Colonies of interest were then successfully isolated through repeated subculturing and were characterised based on colony and cellular morphology, Gram staining and biochemical characterisation with oxidase and catalase activity.

### 4.5. Genomic DNA Extraction from Isolated Microbial Colonies and Phylogenetic Marker Gene Sequencing Targeting Bacterial 16S rRNA and Fungal ITS Regions

For the purpose of identifying microbial populations isolated through various culturing techniques and media compositions, a genomic DNA library was prepared by pooling DNA extracts from the individual isolates within each treatment and were subjected to next generation sequencing with bacterial 16S and fungal ITS gene regions as targets for identification. This approach, as an alternative to Sanger sequencing to identify individual isolates provides a cost-effective assessment of the diversity of isolated microorganisms. Briefly single colonies of each isolate were used to inoculate 5 mL of nutrient broth in a sterile 10 mL tube and incubated for 96 h at 25 °C in a shaking incubator at 220 rpm. One mL of the microbial suspension was transferred to a 1.5 mL microcentrifuge tube and centrifuged at 6000× *g* in a benchtop centrifuge (Eppendorf, HH). DNA extraction was performed on the resulting microbial pellet using a Genelute Bacterial Genomic DNA Kit (NA2120, Sigma Aldrich, NSW) according to the recommended DNA isolation protocol for Gram positive bacteria. 10 µL of each DNA extract was used to produce a composite DNA sample for each treatment group and subjected to phylogenetic marker gene sequencing, performed at the Australian Genome Research Facility (AGRF, QLD). The regions of amplification for sequencing and the primers used are listed in Table 3 [87].

Microbial community diversity analysis was performed following sequencing to determine classes and genera of bacteria/fungi isolated by each microbial isolation method. A limit of >500 sequence reads was used to account for potential polymerase chain reaction (PCR) bias and cross contamination. A subset of the DNA extracts corresponding to a selection of bacterial isolates were subjected to Sanger sequencing for definitive identification (AGRF, VIC).

### 4.6. Genomic DNA Extraction from Rhizosphere Soils

Microbial genomic DNA was extracted from 0.25 g of soil collected and pooled at various growth stages of the canola crop using a Powersoil^®^ DNA Isolation Kit (Mo Bio Laboratories, Carlsbad, CA, USA), according to manufacturer’s instructions. One-hundred microliters of the extracted DNA from each soil sample was sequenced at 16S rRNA and nuclear ribosomal internal transcribed spacer (ITS) regions for bacteria and fungi respectively using an Illumina MiSeq platform as described above.

### 4.7. Extraction of Soil Microbial Metabolites and Metabolic Profiling

Single colonies of pure cultures of selected microorganisms established on nutrient broth solidified with gellan (colony ID 240, 241 and 321- identified as a *Williamsia* sp., a *Rhodococcus* sp. and an *Acinetobacter* sp. respectively) were used to inoculate 20 mL of sterile nutrient broth in 50 mL centrifuge tubes and placed in a shaking incubator at 25 °C for 96 h at 180 rpm in the dark until an optical density of approximately 0.6 was reached. Bacterial and fungal cells were separated from the culture media by centrifugation at 4000 *g* in a benchtop centrifuge. Bacterial cultures from colony IDs 240, 241 and 321 were extracted in 5 mL of a 4:1 mixture of EtOH/ CH_2_Cl_2_. Culture supernatants from 321, 241 and 240 were extracted in 5 mL of a 1:1 mixture of acetone/H_2_O. The solvents were evaporated under nitrogen at 40 °C and the resulting residue resuspended in 5 mL of acetonitrile, filtered using 0.20 µm polytetrafluoroethylene (PTFE) syringe filters (Captiva Econofltr, Agilent Technologies, Mulgrave, VIC, Australia) and stored at 4 °C until UHPLC-DAD-QToF-MS analysis.

Non-targeted metabolic profiling of microbial metabolites in the cellular fractions and culture supernatants of the specific colonies identified above were performed using an Agilent 1290 Infinity UHPLC system equipped with a quaternary pump, diode array detector (DAD), degasser, temperature controlled column (30 °C) and cooled auto-sampler compartments (4 °C) which were coupled to an Agilent 6530 quadrupole time-of-flight (QToF) mass spectrometer (MS) with an Agilent Dual Jet Stream ionisation source (Agilent Technologies, Mulgrave, VIC, Australia). Full scan mass spectra were acquired over a range of 100–3200 Da at a rate of two spectra/second using both positive and negative ion modes. Chromatographic separation was performed in two experimental runs using a reverse phase C18 Poroshell column (2.1 × 100 mm, 2.7 μm particle size) (Agilent Technologies, Santa Clara, CA, USA) connected to a C18 guard column (2.1 × 12.5 mm, 5 μm particle size) (Agilent Technologies, CA, USA) and subsequently to a Kinetex hydrophilic interaction liquid chromatography (HILIC) column (2.1 × 50 mm, 2.6 µm particle size) (Phenomenex, Torrance, CA USA) connected to a HILIC guard column (2.1 × 10 mm, 2.6 µm particle size ). The flow rate of the mobile phase was 0.3 mL/min. The columns were equilibrated for 40 min prior to analysis. Reverse phase chromatographic separation was obtained using a gradient of solvent A (water (Milli-Q, TKA-GenPure), 0.1% formic acid (LC–MS grade, LiChropur^®^, 98–100%, Sigma-Aldrich, Castle Hill, NSW, Australia) and solvent B (95% HPLC-grade acetonitrile (RCI Labscan, Krung Thep Maha Nakhon, Thailand), 0.1% formic acid). The solvent gradient for reverse phase C18 chromatography was 5–100% B in 0–24 min. Separation with the HILIC column employed a gradient of solvent A (10 mM aqueous ammonium acetate and 0.2% formic acid) and solvent B (100% acetonitrile) commencing at 95% B for 0.5 min and decreasing to 35% B at 12.5 min were it remained until 13 min. Ultraviolet absorption was monitored across a range of wavelengths from 210 nm to 640 nm. Mass spectrometry was performed in both positive and negative ion modes with nebulizer gas set at 35 psi, capillary voltage at 3500 V and fragmentor voltage at 135 V. Injection volume was 10 µL for each sample. Nitrogen was used as the drying gas at 250 °C at a flowrate of 9 L/min.

### 4.8. Statistical Analysis

#### 4.8.1. Enumeration and Characterisation of Soil Microbial Cultures

Comparisons between numbers of microbial isolates obtained through modifications to culture conditions and media were analysed using One-way Analysis of Variance (ANOVA) using GraphPad Prism for Mac (Version 7, La Jolla, CA, USA).

#### 4.8.2. Metagenomics Analysis

Data processing and statistical analysis to produce OTU tables was performed by the Australian Genome Research Facility (AGRF). Briefly, paired-end reads were assembled by aligning the forward and reverse reads using PEAR (version 0.95, Exelixis Lab, Heidelberg, Baden-Württemberg, Germany). Primers were identified and trimmed, with the trimmed sequences then processed using Quantitative Insights into Microbial Ecology (QIIME, version 1.8) [91] USEARCH (version 8.0.1623) [92] and UPARSE software [93]. Utilising the tools within USEARCH, sequences were quality filtered, full length duplicate sequences removed and sorted by abundance. Singletons or unique reads in the data set were discarded. Sequences were clustered followed by chimera filtering using “Unite” as the reference database. To obtain number of reads in each OTU, reads were mapped back to OTUs with a minimum identity of 97%. QIIME software was used to assign taxonomy with Unite database as the reference database (Unite Version 7.2) [94] for subsequent generation of absolute abundance values for each OTU.

Rarefaction was performed based on the lowest sample depth using the absolute abundance of samples. Samples for bacteria were rarefied at 30,163 OTUs and fungi was rarefied at 35,972 OTUs across all the samples (Supplementary Materials Figure S4). Analyses of alpha diversity indices (Supplementary Materials Table S3) and relative abundance of each OTU were determined as previously described [95,96]. The OTU relative abundance tables were used to generate graphical representations of taxonomic abundances of bacterial and fungal OTUs present at the phylum level in each experimental group. To assess similar and unique OTUs between the rhizochip and standard aqueous suspension based colony isolation, the OTU abundance data were filtered to represent the number of OTUs present at the family level. Venn diagrams were prepared using Venn Diagram package [97] in R 3.5.2 [98]. SIMPER community analysis [99] was used to assess the contribution of taxa towards observed differences between groups of samples and was performed using Bray-Curtis as the distance/similarity measurement using PAST (Version 3.22) [96].

#### 4.8.3. Metabolic Profiling

A matrix of molecular features characterized by mass to charge ratio (*m*/*z*) and retention time (RT) was generated using Agilent MassHunter Workstation Qualitative software version B07.00, Agilent MassHunter Profinder (version B.08.00), Agilent Mass Profiler Professional (MPP version 14.5) and Agilent Personal Compound Database Library (PCDL) (Agilent Technologies, Santa Clara, CA, USA). The parameters for molecular extracts and peak binning/alignment using Profinder were as follows; peak height ≥ 10,000 counts, compound ion count threshold two or more ions, compound alignment tolerances were 0.00% + 0.15 minutes for RT and 20.00 ppm + 2.00 mDa for mass. The extracted ion chromatogram was smoothed with Gaussian smoothing before integration. The data files were converted to compound exchange files (.cef) format and visualized and analyzed in MPP using multivariate analysis including principal component analysis. Features present in a minimum of three out of five replicates were included for further analysis. Identification was performed comparing the generated database to the METLIN metabolomics database (version B 07.00, Agilent Technologies, Santa Clara, CA, USA) and confirmed using available standards based on accurate mass, retention time (RT) and mass spectra.

## 5. Conclusions

The application and refinement of metabolic profiling using mass spectrometry through the UPLC-DAD-QToF-MS platform applied in this study, along with improved extraction protocols for microbial suspensions, offer promise for the rapid identification of novel microbial metabolites of interest. We believe that the use of an in situ rhizochip prototype to select for enhanced bacterial diversity in the soil rhizosphere warrants additional attention given its success in recovering organisms of high taxonomic diversity, despite lower total numbers recovered, in contrast to the use of more traditional approaches based on inoculation of culture media with aqueous soil suspensions. Collectively the use of both in situ and standard media culture techniques along with both culture dependent and independent methods for isolate identification has facilitated the recovery and identification of novel, rare or slow-growing soil bacteria, and their associated microbial metabolites. Further upscaling of culturing techniques for isolates of interest will allow for enhanced structural elucidation and bioassay of the unique natural products identified by metabolic profiling.

## Figures and Tables

**Table 1:** Identification of a limited group of bacterial colonies of interest by Sanger sequencing.

Colony ID	Source	Identification (Percent Similarity)	Agroecological Relevance	Reference
24	Rhizochip	*Pseudomonas costantinii* (100%)	Pathogen affecting mushrooms	[37]
26	Rhizochip	*Pseudomonas* sp. str. LaGso27l (98.7%)		
32	Rhizochip	*Paenibacillus polymyxa* str. BMP-11 (100%)	Insecticidal and herbicidal activity	[38]
37	Rhizochip	*Chryseobacterium* sp. str. KR200 (100%)		
45	Rhizochip	*Chryseobacterium indologenes* str. H2S10 (100%)		
238	MH	*Arthrobacter nitroguajacolicus* str. G2-1 (100%)	4-nitroguaiacol-degradation	[39]
240	MH	*Williamsia muralis* str. 9571414 (99.9%)		
241	MH	*Rhodococcus sp.* str. 5/14 (99.85%)		
321	NG	*Acinetobacter sp.* (100%)	Model species for environmental and biotechnological applications	[40]
343	MH	*Brevibacillus laterosporus* str. BL-2 (99.86%)	Invertebrate pathogen	[41]
354	NG	*Variovorax paradoxus* str. rif200835 (99.72%)	Plant growth promotion	[42,43,44,45]
362	NG	*Variovorax* sp. A2 (100%)		
364	NG	*Arthrobacter nicotinovorans* (100%)	Herbicide degradation (Atrazine)	[46]
365	NG	*Rhodococcus* sp. str. 5/14 (99.71%)		

NG: nutrient broth gellan; MH: Mueller Hinton.

**Table 2:** Soil sampling dates corresponding to the growth stage of canola.

Sampling Date	Growth Stage of Canola Crop
18 May 2016	planting
11 July 2016	pre-flowering
6 October 2016	flowering
7 November 2016	post-flowering
8 December 2016	harvest

**Table 3:** Primers used for Illumina Hiseq application.

Primer Name	Primer Sequence	Reference
27F—Universal 16S	AGAGTTTGATCMTGGCTCAG	[88]
519R—Universal 16S	GWATTACCGCGGCKGCTG	[88]
1F—Universal ITS	CTTGGTCATTTAGAGGAAGTAA	[89]
2R—Universal ITS	TGTGTTCTTCATCGATG	[90]

## Supplementary Materials

The following are available online at https://www.mdpi.com/1420-3049/24/10/1955/s1, Table S1: Bray Curtis dissimilarity indices for Rhizochip, Nutrient broth gellan and Mueller Hinton isolates, Table S2: Microbial isolates identified through phylogenetic marker gene sequencing targeting bacterial 16S rRNA region, Table S3: Alpha diversity indices for bacterial and fungal OTUs in rhizosphere soil at various growth stages, Figure S1: Heatmaps summarising the variation in the composition of rhizosphere bacterial (A) and fungal (B) communities at various growth stages of crop growth, Figure S2: Impact of media selection and incubation time of soil suspension on CFUs, Figure S3: Heatmap summarising the composition of bacterial isolates obtained through isolation with the rhizochip and standard culture media, Figure S4: Rarefaction analysis for rhizosphere soil at various stages of crop growth for bacteria (**A**) and fungi (**B**) at 97% sequence similarity.
