# Supplementary material for: Technologies for the Selection, Culture and Metabolic Profiling of Unique Rhizosphere Microorganisms for Natural Product Discovery
Source: Molecules. 2019 May 21;24(10):1955. doi: 10.3390/molecules24101955 (PMC6571749; doi:10.3390/molecules24101955)
Supplement: Supplementary file 1 [file molecules-24-01955-s001.pdf]

Supplementary Information

Table S1. Bray Curtis dissimilarity indices for Rhizochip, Nutrient broth/ gellan and Mueller Hinton isolates

**Bray Curtis dissimilarity index**			
**A. Nutrient broth/ gellan vs Mueller Hinton**			
**Taxon**	**Average dissimilarity (%)**	**Contribution (%)**	**Cumulative percentage (%)**
Bacillales	7.114	14.37	14.37
Clostridiales	7.104	14.35	28.73
Xanthomonadales	3.358	6.785	35.51
Bacteroidales	2.699	5.453	40.97
Pseudomonadales	2.54	5.132	46.1
Actinomycetales	2.245	4.536	50.63
Enterobacteriales	2.245	4.536	55.17
Burkholderiales	2.19	4.425	59.59
Rhizobiales	2.045	4.132	63.73
Sphingobacteriales	1.651	3.337	67.06
Coriobacteriales	1.651	3.337	70.4
Gaiellales	1.567	3.165	73.57
RF39	1.271	2.569	76.13
Rubrobacterales	1.271	2.569	78.7
AKYG885	1.258	2.542	81.24
Verrucomicrobiales	1.258	2.542	83.79
Erysipelotrichales	1.211	2.446	86.23
Turicibacterales	0.9733	1.967	88.2
Solirubrobacterales	0.9733	1.967	90.17
Lactobacillales	0.9733	1.967	92.13
Anaeroplasmatales	0.9733	1.967	94.1
Caulobacterales	0.9733	1.967	96.07
Actinomycetales;Other;Other;Other	0.9733	1.967	98.03
0319-7L14	0.9733	1.967	100
Nitrospirales	0	0	100
Flavobacteriales	0	0	100
Acidithiobacillales	0	0	100
**B. Rhizochip vs Mueller Hinton**			
Actinomycetales	6.673	14.77	14.77
Bacteroidales	5.499	12.17	26.94
Clostridiales	5.078	11.24	38.18
Bacillales	3.72	8.234	46.42
Pseudomonadales	2.665	5.899	52.31
Burkholderiales	2.172	4.806	57.12
Erysipelotrichales	2.143	4.743	61.86
Gaiellales	1.729	3.828	65.69
Xanthomonadales	1.701	3.765	69.46

Enterobacteriales	1.377	3.047	72.5
Flavobacteriales	1.271	2.814	75.32
0319-7L14	1.271	2.814	78.13
Verrucomicrobiales	1.253	2.773	80.9
Rhizobiales	1.253	2.773	83.68
AKYG885	1.253	2.773	86.45
Nitrospirales	1.224	2.71	89.16
Actinomycetales;Other;Other;Other	1.224	2.71	91.87
Acidithiobacillales	1.224	2.71	94.58
Rubrobacterales	1.224	2.71	97.29
Caulobacterales	1.224	2.71	100
**C. Rhizochip vs Nutrient broth/ gellan**			
Bacillales	5.306	11.27	11.27
Actinomycetales	4.802	10.2	21.47
Bacteroidales	3.903	8.289	29.76
Clostridiales	3.217	6.832	36.59
Pseudomonadales	3.197	6.791	43.38
Xanthomonadales	2.714	5.764	49.14
Erysipelotrichales	2.072	4.4	53.54
Rhizobiales	1.895	4.026	57.57
Enterobacteriales	1.696	3.601	61.17
Burkholderiales	1.412	2.999	64.17
Rubrobacterales	1.4	2.974	67.14
Gaiellales	1.382	2.935	70.08
0319-7L14	1.338	2.842	72.92
Actinomycetales;Other;Other;Other	1.318	2.8	75.72
Caulobacterales	1.318	2.8	78.52
Sphingobacteriales	1.302	2.765	81.29
Coriobacteriales	1.302	2.765	84.05
RF39	1.054	2.239	86.29
Flavobacteriales	1.052	2.235	88.52
Nitrospirales	1.019	2.165	90.69
Acidithiobacillales	1.019	2.165	92.85
Turicibacterales	0.8412	1.787	94.64
Solirubrobacterales	0.8412	1.787	96.43
Lactobacillales	0.8412	1.787	98.21
Anaeroplasmatales	0.8412	1.787	100

Table S2. Microbial isolates identified through phylogenetic marker gene sequencing targeting bacterial 16S rRNA region in canola rhizosphere soil collected at crop harvest.

Isolated taxa	Method(s) of isolation	Taxonomy
0319-7L14	RC/NG	Bacteria∣Actinobacteria∣MB-A2-108∣0319-7L14
Acidimicrobiales	NG	Bacteria∣Actinobacteria∣Acidimicrobiia∣Acidimicrobiales
Acidithiobacillus	RC	Bacteria∣Proteobacteria∣Gammaproteobacteria∣Acidithiobacillales∣Acidithiobacillaceae∣Acidithiobacillus
Actinomycetales∣Other∣Other	RC/NG	Bacteria∣Actinobacteria∣Actinobacteria∣Actinomycetales
Actinomycetales	RC	Bacteria∣Actinobacteria∣Actinobacteria∣Actinomycetales
Agrobacterium	NG	Bacteria∣Proteobacteria∣Alphaproteobacteria∣Rhizobiales∣Rhizobiaceae∣Agrobacterium
AKYG885	MH	Bacteria∣Chloroflexi∣TK10∣AKYG885
Alcaligenaceae	RC/NG/MH	Bacteria∣Proteobacteria∣Betaproteobacteria∣Burkholderiales∣Alcaligenaceae
Allobaculum	RC/MH	Bacteria∣Firmicutes∣Erysipelotrichi∣Erysipelotrichales∣Erysipelotrichaceae∣Allobaculum
Anaeroplasmataceae	NG	Bacteria∣Tenericutes∣Mollicutes∣Anaeroplasmatales∣Anaeroplasmataceae
Aureus	NG/MH	Bacteria∣Firmicutes∣Bacilli∣Bacillales∣Staphylococcaceae∣Staphylococcus∣Aureus
Bacillaceae	NG/MH	Bacteria∣Firmicutes∣Bacilli∣Bacillales∣Bacillaceae
Bacillus	RC/NG/MH	Bacteria∣Firmicutes∣Bacilli∣Bacillales∣Bacillaceae∣Bacillus
Bacteroides∣Other	RC/NG/MH	Bacteria∣Bacteroidetes∣Bacteroidia∣Bacteroidales∣Bacteroidaceae∣Bacteroides∣Other
Bacteroides	RC/NG/MH	Bacteria∣Bacteroidetes∣Bacteroidia∣Bacteroidales∣Bacteroidaceae∣Bacteroides
Barnesiellaceae	RC	Bacteria∣Bacteroidetes∣Bacteroidia∣Bacteroidales∣Barnesiellaceae
Blautia	RC	Bacteria∣Firmicutes∣Clostridia∣Clostridiales∣Lachnospiraceae∣Blautia
Brevibacillus∣Other	RC/NG	Bacteria∣Firmicutes∣Bacilli∣Bacillales∣Paenibacillaceae∣Brevibacillus∣Other
Caulobacteraceae	RC/NG	Bacteria∣Proteobacteria∣Alphaproteobacteria∣Caulobacterales∣Caulobacteraceae
Cereus	RC/NG/MH	Bacteria∣Firmicutes∣Bacilli∣Bacillales∣Bacillaceae∣Bacillus∣ Cereus
Chryseobacterium	RC	Bacteria∣Bacteroidetes∣Flavobacteriia∣Flavobacteriales∣Weeksellaceae∣Chryseobacterium
Clostridiaceae	NG/MH	Bacteria∣Firmicutes∣Clostridia∣Clostridiales∣ Clostridiaceae
Clostridiales	RC/NG	Bacteria∣Firmicutes∣Clostridia∣Clostridiales
Clostridium	NG/MH	Bacteria∣Firmicutes∣Clostridia∣Clostridiales∣Clostridiaceae∣Clostridium
Aerofaciens	NG	Bacteria∣Actinobacteria∣Coriobacteriia∣Coriobacteriales∣Coriobacteriaceae∣Collinsella∣Aerofaciens
Copri	NG	Bacteria∣Bacteroidetes∣Bacteroidia∣Bacteroidales∣Prevotellaceae∣Prevotella∣Copri
Enterobacteriaceae	RC/NG	Bacteria∣Proteobacteria∣Gammaproteobacteria∣Enterobacteriales∣Enterobacteriaceae∣Other∣Other
Erysipelotrichaceae	RC	Bacteria∣Firmicutes∣Erysipelotrichi∣Erysipelotrichales∣Erysipelotrichaceae

Fascians	RC	Bacteria∣Actinobacteria∣Actinobacteria∣Actinomycetales∣Nocardiaceae∣Rhodococcus∣Fascians
Flexus	RC/MH	Bacteria∣Firmicutes∣Bacilli∣Bacillales∣Bacillaceae∣Bacillus∣ Flexus
Gaiellaceae	RC/NG/MH	Bacteria∣ Actinobacteria∣ Thermoleophilia∣Gaiellales∣Gaiellaceae∣Other∣Other
Gluconacetobacter	NG	Bacteria∣Proteobacteria∣Gammaproteobacteria∣Enterobacteriales∣Enterobacteriaceae∣Gluconacetobacter
Lachnospiraceae	RC/NG/MH	Bacteria∣Firmicutes∣Clostridia∣Clostridiales∣Lachnospiraceae
Lachnospiraceae∣Other∣Other	RC/NG	Bacteria∣Firmicutes∣Clostridia∣Clostridiales∣Lachnospiraceae∣Other∣Other
Lactobacillus	NG	Bacteria∣Firmicutes∣Bacilli∣Lactobacillales∣Lactobacillaceae∣Lactobacillus
Lautus	NG/MH	Bacteria∣Firmicutes∣Bacilli∣Bacillales∣Paenibacillaceae∣Paenibacillus∣Lautus
Leptospirillum	RC	Bacteria∣Nitrospirae∣Nitrospira∣Nitrospirales∣Leptospirillaceae∣Leptospirillum
Leucobacter	RC	Bacteria∣Actinobacteria∣Actinobacteria∣Actinomycetales∣Microbacteriaceae∣Leucobacter
Lysinibacillus∣Boronitolerans	RC/NG/MH	Bacteria∣Firmicutes∣Bacilli∣Bacillales∣Planococcaceae∣Lysinibacillus∣Boronitolerans
Megamonas	RC/NG	Bacteria∣Firmicutes∣Clostridia∣Clostridiales∣Veillonellaceae∣Megamonas
Microbacterium	RC	Bacteria∣Actinobacteria∣Actinobacteria∣Actinomycetales∣Microbacteriaceae∣Microbacterium
Microbacterium∣Other	RC	Bacteria∣Actinobacteria∣Actinobacteria∣Actinomycetales∣Microbacteriaceae∣Microbacterium∣Other
Micrococcaceae∣Other∣Other	NG	Bacteria∣Actinobacteria∣Actinobacteria∣Actinomycetales∣Micrococcaceae
Micrococcaceae	NG	Bacteria∣Actinobacteria∣Actinobacteria∣Actinomycetales∣Micrococcaceae
Moraxella	RC/NG/MH	Bacteria∣Proteobacteria∣Gammaproteobacteria∣Pseudomonadales∣Moraxellaceae∣Moraxella
Moraxellaceae	RC	Bacteria∣Proteobacteria∣Gammaproteobacteria∣Pseudomonadales∣Moraxellaceae
Muciniphila	MH	Bacteria∣Verrucomicrobia∣Verrucomicrobiae∣Verrucomicrobiales∣Verrucomicrobiaceae∣Akkermansia∣ Muciniphila
Multivorum	NG/MH	Bacteria∣Bacteroidetes∣Sphingobacteriia∣Sphingobacteriales∣Sphingobacteriaceae∣Sphingobacterium∣Multivorum
Muralis	RC/NG/MH	Bacteria∣Firmicutes∣Bacilli∣Bacillales∣Bacillaceae∣Bacillus∣ Muralis
Oscillospira	NG/MH	Bacteria∣Firmicutes∣Clostridia∣Clostridiales∣Ruminococcaceae∣Oscillospira
Paenibacillus	RC/MH	Bacteria∣Firmicutes∣Bacilli∣Bacillales∣Paenibacillaceae∣Paenibacillus
Paenibacillus∣Other	RC/NG/MH	Bacteria∣Firmicutes∣Bacilli∣Bacillales∣Paenibacillaceae∣Paenibacillus∣Other
Paradoxus	MH	Bacteria∣Proteobacteria∣Betaproteobacteria∣Burkholderiales∣Comamonadaceae∣Variovorax∣Paradoxus
Phascolarctobacterium	RC	Bacteria∣Firmicutes∣Clostridia∣Clostridiales∣Veillonellaceae∣Phascolarctobacterium
Phyllobacteriaceae	NG/MH	Bacteria∣Proteobacteria∣Alphaproteobacteria∣Rhizobiales∣Phyllobacteriaceae
Reuszeri	NG/MH	Bacteria∣Firmicutes∣Bacilli∣Bacillales∣Paenibacillaceae∣Brevibacillus∣Reuszeri

RF39	NG	Bacteria|Tenericutes|Mollicutes|RF39
Roseburia	RC	Bacteria|Firmicutes|Clostridia|Clostridiales|Lachnospiraceae|Roseburia
Rubrobacter	RCN/G	Bacteria|Actinobacteria|Rubrobacteria|Rubrobacterales|Rubrobacteraceae|Rubrobacter
Ruminococcaceae|Other|Other	RC/NG	Bacteria|Firmicutes|Clostridia|Clostridiales|Ruminococcaceae|Other|Other
Ruminococcaceae	NG/MH	Bacteria|Firmicutes|Clostridia|Clostridiales|Ruminococcaceae
Ruminococcus	NG	Bacteria|Firmicutes|Clostridia|Clostridiales|Ruminococcaceae|Ruminococcus
S24-7	RC	Bacteria|Bacteroidetes|Bacteroidia|Bacteroidales|S24-7
Solirubrobacterales	NG	Bacteria|Actinobacteria|Thermoleophilia|Solirubrobacterales
Staphylococcus	RC/NG/MH	Bacteria|Firmicutes|Bacilli|Bacillales|Staphylococcaceae|Staphylococcus
Stenotrophomonas	RC/NG/MH	Bacteria|Proteobacteria|Gammaproteobacteria|Xanthomonadales|Xanthomonadaceae|Stenotrophomonas
Stenotrophomonas|Other	RC/MH	Bacteria|Proteobacteria|Gammaproteobacteria|Xanthomonadales|Xanthomonadaceae|Stenotrophomonas|Other
Sulfobacillus	RC	Bacteria|Firmicutes|Clostridia|Clostridiales|Sulfobacillaceae|Sulfobacillus
Turicibacter	NG	Bacteria|Firmicutes|Bacilli|Turicibacterales|Turicibacteraceae|Turicibacter
Uniformis	RC/NG	Bacteria|Bacteroidetes|Bacteroidia|Bacteroidales|Bacteroidaceae|Bacteroides|Uniformis
Veronii	RC/NG	Bacteria|Proteobacteria|Gammaproteobacteria|Pseudomonadales|Pseudomonadaceae|Pseudomonas|Veronii
Xanthomonadaceae	RC/NG/MH	Bacteria|Proteobacteria|Gammaproteobacteria|Xanthomonadales|Xanthomonadaceae
Xanthomonadaceae|other|other	RC/NG/MH	Bacteria|Proteobacteria|Gammaproteobacteria|Xanthomonadales|Xanthomonadaceae|other|other

RC, Rhizochip; NG, Nutrient broth gellan; MH, Mueller Hinton

Table S3. Alpha diversity indices for bacterial and fungal OTUs in canola rhizosphere soil at various growth stages

Diversity index	Planting	Pre-flowering	Flowering	Post-flowering	Post-harvest
**Bacteria**					
Shannon	7.149	6.136	4.832	7.227	6.979
Evenness	0.3364	0.1645	0.1449	0.3985	0.2817
**Fungi**					
Shannon	3.957	2.741	3.123	4.164	3.38
Evenness	0.1304	0.05988	0.07836	0.1496	0.07687

A

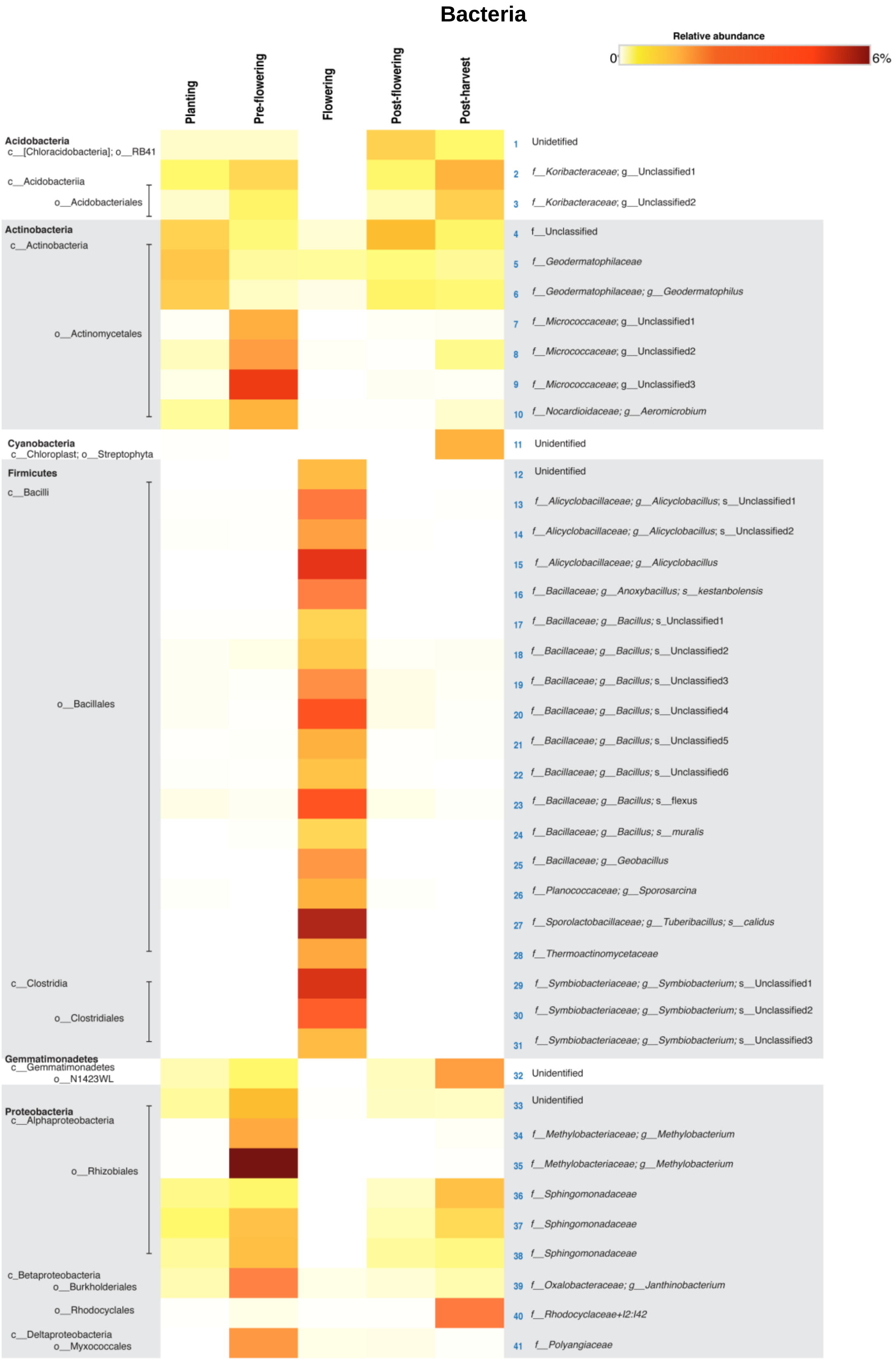

Bacteria
Relative abundance
0
6%
Planting
Pre-flowering
Flowering
Post-flowering
Post-harvest
Acidobacteria
c__[Chloracidobacteria]; o__RB41
c__Acidobacteriia
o__Acidobacteriales
Actinobacteria
c__Actinobacteria
o__Actinomycetales
Cyanobacteria
c__Chloroplast; o__Streptophyta
Firmicutes
c__Bacilli
o__Bacillales
c__Clostridia
o__Clostridiales
Gemmatimonadetes
c__Gemmatimonadetes
o__N1423WL
Proteobacteria
c__Alphaproteobacteria
o__Rhizobiales
c_Betaproteobacteria
o__Burkholderiales
o__Rhodocyclales
c__Deltaproteobacteria
o__Myxococcales
1 Unidetified
2 f__Koribacteraceae; g__Unclassified1
3 f__Koribacteraceae; g__Unclassified2
4 f__Unclassified
5 f__Geodermatophilaceae
6 f__Geodermatophilaceae; g__Geodermatophilus
7 f__Micrococcaceae; g__Unclassified1
8 f__Micrococcaceae; g__Unclassified2
9 f__Micrococcaceae; g__Unclassified3
10 f__Nocardioidaceae; g__Aeromicrobium
11 Unidentified
12 Unidentified
13 f__Alicyclobacillaceae; g__Alicyclobacillus; s__Unclassified1
14 f__Alicyclobacillaceae; g__Alicyclobacillus; s__Unclassified2
15 f__Alicyclobacillaceae; g__Alicyclobacillus
16 f__Bacillaceae; g__Anoxybacillus; s__kestanbolensis
17 f__Bacillaceae; g__Bacillus; s_Unclassified1
18 f__Bacillaceae; g__Bacillus; s__Unclassified2
19 f__Bacillaceae; g__Bacillus; s__Unclassified3
20 f__Bacillaceae; g__Bacillus; s__Unclassified4
21 f__Bacillaceae; g__Bacillus; s__Unclassified5
22 f__Bacillaceae; g__Bacillus; s__Unclassified6
23 f__Bacillaceae; g__Bacillus; s__flexus
24 f__Bacillaceae; g__Bacillus; s__muralis
25 f__Bacillaceae; g__Geobacillus
26 f__Planococcaceae; g__Sporosarcina
27 f__Sporolactobacillaceae; g__Tuberibacillus; s__calidus
28 f__Thermoactinomycetaceae
29 f__Symbiobacteriaceae; g__Symbiobacterium; s__Unclassified1
30 f__Symbiobacteriaceae; g__Symbiobacterium; s__Unclassified2
31 f__Symbiobacteriaceae; g__Symbiobacterium; s__Unclassified3
32 Unidentified
33 Unidentified
34 f__Methylobacteriaceae; g__Methylobacterium
35 f__Methylobacteriaceae; g__Methylobacterium
36 f__Sphingomonadaceae
37 f__Sphingomonadaceae
38 f__Sphingomonadaceae
39 f__Oxalobacteraceae; g__Janthinobacterium
40 f__Rhodocyclaceae+I2:I42
41 f__Polyangiaceae

B

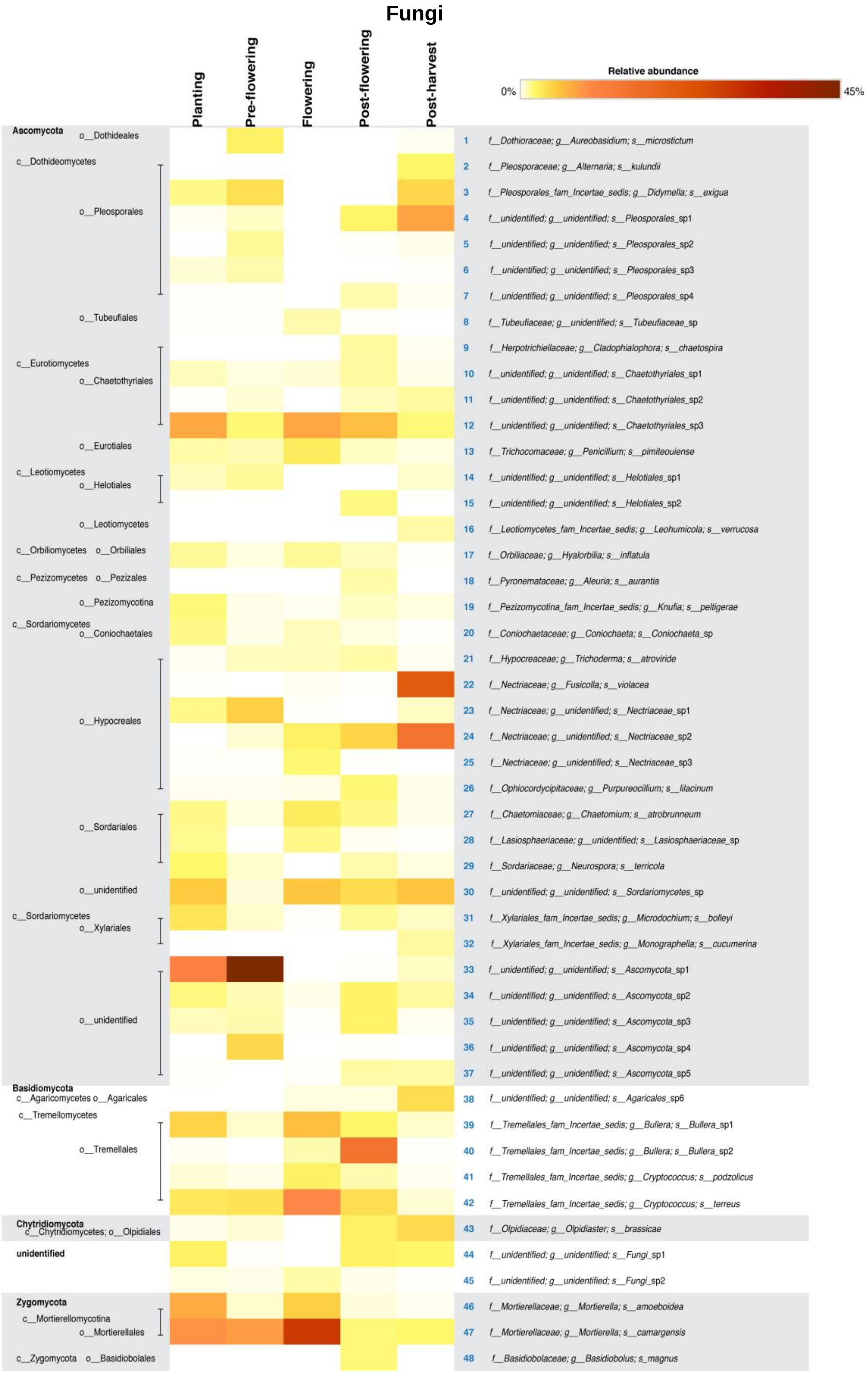

Figure S1. Heatmaps summarising the variation in the composition of rhizosphere bacterial (A) and fungal (B) communities at various growth stages of canola crop growth. OTUs listed are those present at ≥1 % relative abundance at each sampling time.

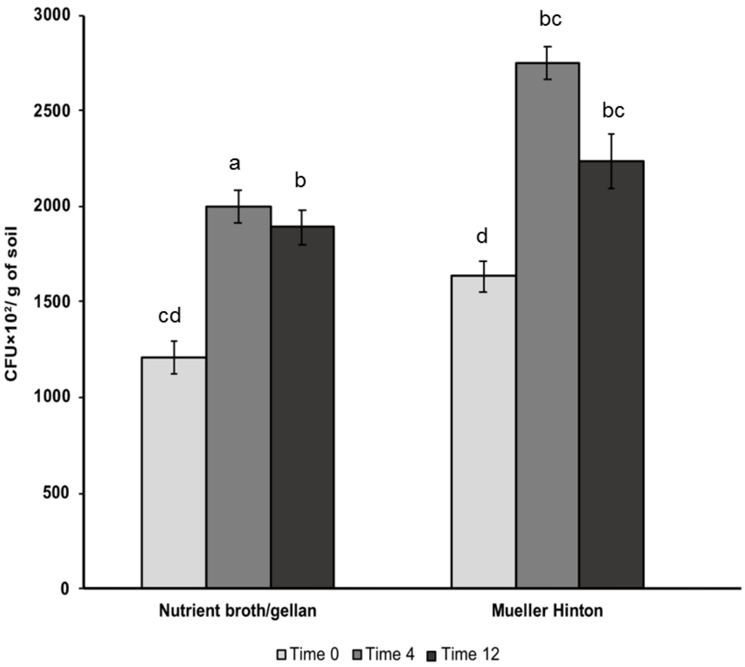

Figure S2. Impact of media selection and incubation time of aqueous soil suspension collected from canola rhizosphere soil at crop harvest on CFUs . Means sharing the same letters are not significantly different.

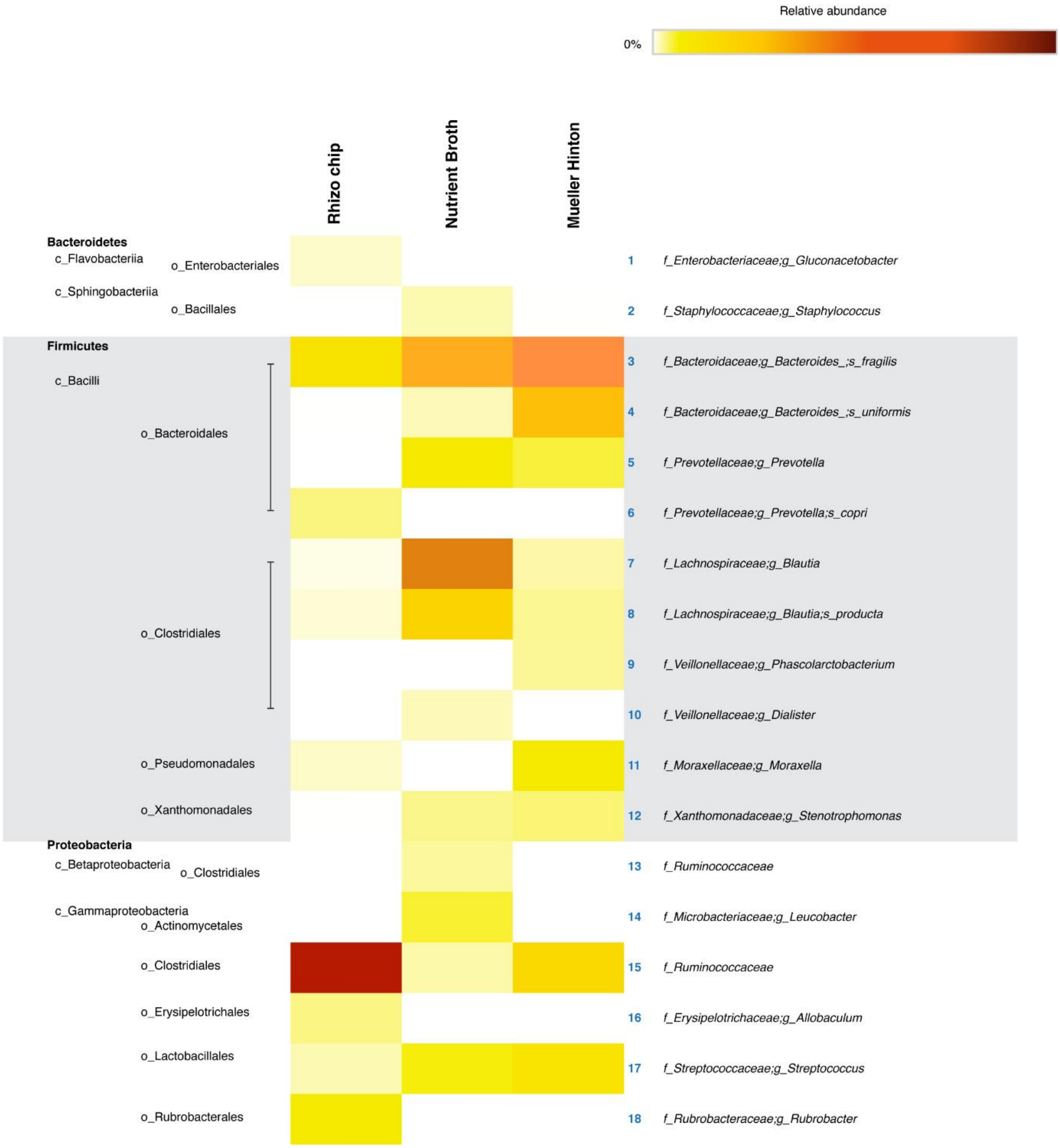

Figure S3. Heatmap summarising the composition of bacterial isolates obtained through isolation from canola rhizosphere soil at harvest using the rhizochip and two standard culture media. OTUs listed are those present at ≥1 % relative abundance.

A

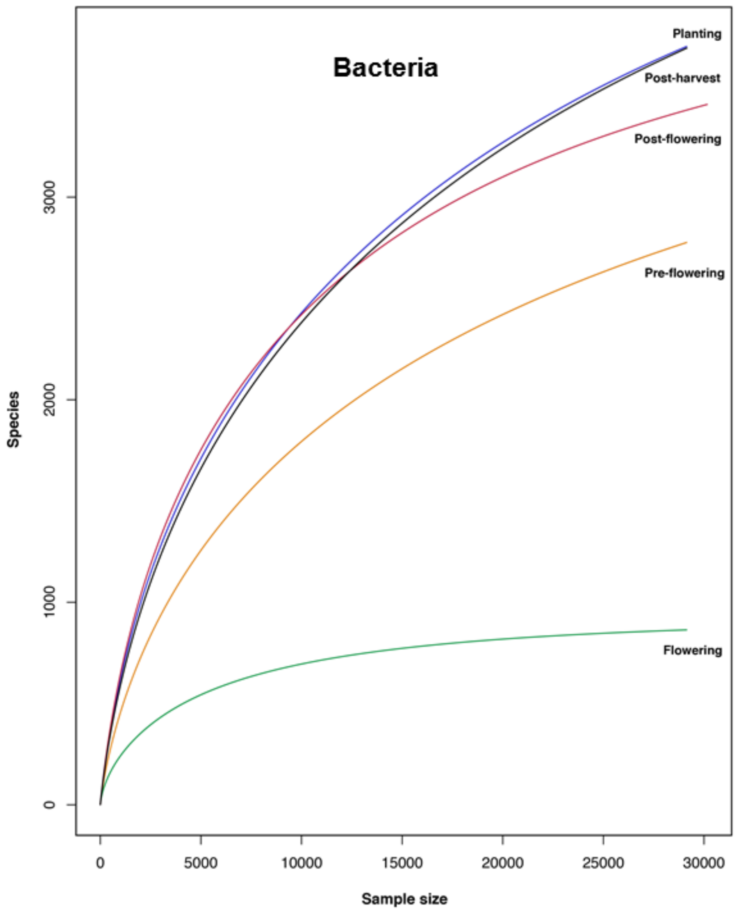

B

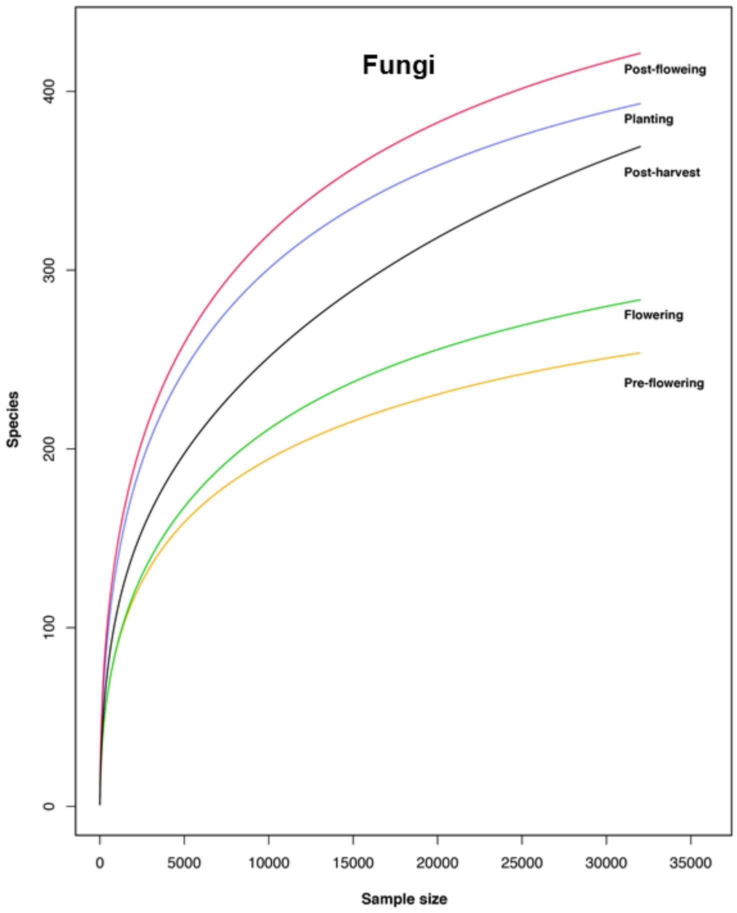

Figure S4. Rarefaction analysis for canola rhizosphere soil at various stages of crop growth for bacteria (A) and fungi (B) at 97% sequence similarity.
